# Social Media Support and Funding Assistance for Psychological Injuries in Social Work

**DOI:** 10.3389/fpubh.2022.796769

**Published:** 2022-05-04

**Authors:** Xiaowu Hu, Caiyi Tang, Dongmei Wang

**Affiliations:** ^1^Department of Social Work and Social Policy, School of Social and Behavioral Sciences, Nanjing University, Nanjing, China; ^2^Department of Psychology, School of Social and Behavioral Sciences, Nanjing University, Nanjing, China

**Keywords:** psychological injury, social work, social media, funding assistance, social marketing

## Abstract

**Introduction:**

Psychological injuries in social work are on the rise in complex modern society. Some individuals are incurring both physical and psychological injuries. Often, psychological injuries are more miserable than physical injuries. To combat the psychological injury suffered by individuals involved in social work, authorities should mobilize support via social media and raise funds by this and other feasible means to cover the cost of care for these individuals. This study focuses on social media support and funding assistance that could play useful roles in helping to treat psychological injuries among social workers and their clients in China.

**Methods:**

A scoping review of academic and gray literature was undertaken to identify the different injuries involved in social work. Semi-structured interviews were carried out with 7 experts, including social workers, social media professionals, and social fund directors. Empirical studies on psychological injuries in social work provided examples in support of the policy advocacy reported in this paper.

**Results:**

The scoping review found diverse literature on the subject of psychological injury in social work over the past decade in China. Semi-structured interviews with experts indicate that social media support can alleviate psychological suffering and that funding assistance has a positive influence on assisting individuals coping with psychological injuries. The empirical cases support the plan to encourage more support from social media and funding sources.

**Conclusion:**

Psychological injury is greatly influenced by social bias and discrimination. According to cases and actions are taken to mitigate the harm done, supportive social media strategies could greatly diminish the psychological injuries to social workers and their clients and help them avoid much suffering. This study finds that funding organizations could provide a new treatment mechanism—social media marketing strategies and functional activities—to help a large number of individuals with psychological injuries out of the disease trap in China.

## Introduction

There is inevitably an increase in the sum of psychological harm in social work practice worldwide. In these suffering groups, there were children, adolescents, elderly individuals, college students, grassroots workers, vocational staff, and social workers. Trauma often occurs after a great change in living conditions or society, especially unexpected social and natural disasters such as an earthquake, flood, infectious disease, or serious transportation accident. According to the literature, there are a great many individuals with resulting psychological damage involved as caregivers and clients in social work. More than 100 million individuals in China suffer from psychological trauma. Understanding the different causes of psychological injury groups is the first step toward taking actions to help them.

In China, most of the children who suffer psychological injury are migrants, who are adversely affected by the processes of urbanization and modernization, as they are forced to change their living environment. Further, while under pressure to conform to their new lifestyles, migrant children cannot often communicate with their new urban peers (1). Another group of children who are vulnerable to psychological harm is left-behind children in rural areas. Because of the acceleration of urbanization, parents often leave their children behind with relatives while they seek employment in urban centers. These children lack the care and guidance of their parents, and their emotional needs, such as attachment and communication, cannot be met. There is a sense of alienation between these children and their parents. Xie Jiandu and Cai Xiaodong found that left-behind children generally exhibit inferiority, autism, depression, introversion, and low self-esteem (2). Cao Ziai, Wudi, and others believe that left-behind children have self-cognition problems, interpersonal relationship issues, and negative personality traits that compromise their mental health (3). In addition, among the children who have suffered psychological harm are those in orphanages and children with congenital diseases.

The mental health and psychological problems of adolescents are mainly concentrated in three aspects: family, peer groups, and cyberspace. Peer groups have a very important impact on the mental health of young people. Individuals with psychological issues cannot handle relationships among peer groups very well. They are prone to physical cognitive biases, a lack of self-confidence and self-efficacy, and even school weariness and persistent violent attacks on campus. Zhang Yingying and Zeng Yu believe that the negative emotions generated in peer-group relationships can cause school maladjustment, high-risk behaviors, and behavioral disorders among teenagers and may lead to emotional problems such as social anxiety, social depression, social fear, and loneliness (4). Families play an important and irreplaceable role in the growth of adolescents. Li Zhenpeng believes that most single parents usually cannot provide good support for the psychological development of youth, so these children are challenged in the practice of constructing self-identity, and they will have psychological deviations because of the loneliness and depression in their daily lives (5). Many adolescents have active online lives, but social lives conducted via the internet can have harmful effects on young people because of cheating, false advertisements, and emotional deception that those they communicate with can perpetuate. Thus, many adolescents suffer psychological injury from internet addiction, abusive interactions, and a sense of inferiority generated by the lives of others they see online.

In social work, many elderly individuals are seen for psychological injury due to physical disease, loneliness, and social exclusion. China's population is rapidly aging. The mismatch between demand for services and dashed expectations has led to a series of psychological problems for elderly individuals seeking help. Yang Ling, Jin Xiafang, and others believe that age is the source of many psychological problems such as loneliness, worry, and anxiety (6). Zhou H's team found that elderly individuals living in nursing homes are more psychologically negative (7).

It is a new phenomenon in social work practice that an increasing number of college students are reporting psychological distress, and the incidence of suicides is on the rise in many places. The pressures of schoolwork, academic competition, personal choices and worries, complicated interpersonal relationships, conflicts, and failures in love all-cause college students to have serious physical and mental health problems. Related survey data show that the mental health of college students in China has increased rapidly. A considerable number of colleges report negative and stressful environments. College students who drop out due to mental illness account for more than 50% of the total number of dropouts. Psychological problems have become an important factor affecting the individual development of college students and the stability of their schools (8).

Even grassroots workers and vocational staff are currently experiencing psychological anxiety. The fast tempo of the contemporary marketplace and changing values are pushing blue-collar and white-collar workers into a fierce competitive life stage and social comparison (9). Anxiety from work, life, interpersonal relationships, and career prospects have led to growing psychological stress among grassroots workers and vocational staff, such as technical personnel, doctors, nurses, teachers, professors, judges, social workers, and therapists. Although these professionals have a high degree of social recognition, the pressure of their responsibilities is too high, and social support is insufficient, which leads to a decrease in self-efficacy and an increase in psychological problems (10). Enduring emotional pressure and lacking social support have resulted in job burnout, low subjective well-being, huge work anxiety, and psychological trauma.

Meanwhile, due to the outbreak of Coronavirus Disease 2019 (COVID-19), millions of people have experienced new psychological harm as a consequence of the virus and anti-viral measures, including home quarantine, isolation, and physical illness while they may also incur a loss of income. In addition to disaster-stricken people, groups such as medical workers and social workers who participate in the rescue of others will also be affected by overwork, reduced social support, and exceeding expectations in the process of rescue and assistance. Under pressure, negative psychological emotions such as fear, anxiety, tension, grief and helplessness, depression, and sadness occur (11).

Indeed, there is a large number of psychological injuries found in social work practice in China at the time of fast urbanization, high competitiveness, the transition to a market economy, and individual achievement. Therefore, we should find practical strategies and take effective actions to address this problem. In this study, we aim to conduct a scoping review of the published articles and interview 7 experts to identify the roles of social media support and funding assistance in helping to treat psychological injuries among social workers and their clients in China. Furthermore, A new mechanism of social work on psychological injury is proposed.

## Materials and Methods

### Scoping Review of Literature

A scoping review of the existing academic and gray literature was undertaken to examine the traditional social work actions taken to address psychological harm. We found that social work clients may be categorized as case work, group work, or community work and that individuals from different backgrounds had different psychological problems. Semi-structured interviews provided more information and inspiration for practical measures to resolve psychological harm.

Case work has a very prominent role in improving psychological health. It started at the end of World War I in response to the worldwide economic crisis in 1929. The strong influence of German psychoanalytic psychology helped case work begin to use psychoanalytic methods to help recipients solve problems, and it formed the characteristics of focusing on personal psychological factors rather than social environment (12). Case work on psychological injury is mainly based on the therapy of psychological clinics and hospitals. This mainly focuses on the psychological aspect, and the concept of “people in the environment” is tantamount to targeting different life stages, such as children, adolescents, and elderly individuals, and groups in different environments, such as migrant children, poor college students, disaster-affected people, and patients with diseases. This method also gives corresponding solutions to psychological problems. Regarding the six groups mentioned in the first part, we can appreciate the uniqueness of case work to each vulnerable group. The greatest advantage of case work is that it can solve problems from person to person. At the same time, the client can respond in a relatively short period. For example, after the outbreak of COVID-19, the most successful way to intervene in the burnout of medical staff was case work (13). Correspondingly, because of its strong pertinence and individuality, case work has the disadvantages of being time-consuming, high cost, and low impact. Therefore, case work is limited in China due to the large population.

Group work is helpful and practical when dealing with psychological injury. Based on psychoanalytic theory, the group work method has effective functions for common psychological activities in groups, especially teenagers. Teenagers often cluster because they are afraid of loneliness, and they pursue identity and other developmental characteristics. Group work has the advantage of solving the psychological problems of adolescents while meeting their needs for peer recognition. It focuses on helping group members form a mutual aid partnership to obtain high cohesion and a sense of security so they can express their feelings and thoughts more openly. However, excessive pursuit of organizational strength will inevitably neglect individual needs. Therefore, cultivating group cohesion is an essential and important part of group work, but there will still be insufficient assessments of team members' awareness and the degree of psychological change in group work, which will lead to unclear progress in personal transformation and resocialization (14).

Community work is a comparatively new method to address psychological harm. There is an increasing number of special groups, such as “left-behind children” and “empty nesters”, in the rapid urbanization period in modern China (15). This prompts community work to play an important role in addressing the new psychological problems of clients produced by uneven urbanization in China. Community social work focuses on changes in the macrosocial environment and ecological system to relieve the burdens of clients who lack opportunities and resources. However, community social work requires the innovation of social policy and an institutional supply of resources, and it is a long and slow method to cope with the urgent problem of psychological injuries in children, elderly individuals, and other groups who are suffering from the impact of urbanization and aging.

### Semi-structured Interviews

In this study, we interviewed 7 experts from different institutions including social workers, social media professionals, and social fund directors. A summary of the interviewees' information is listed here: A, social worker; B, psychiatrist; C, university psychological counselor; D, professor of social work; E, media thinktank expert; F, an official of Health Commission; G, professor of social policy. The 7 interviewees were interviewed informally following a rough outline of the interview. Three questions are asked like this: Q1: Why there is increasing growth of psychological injuries? Q2: How can we help a large number of psychological injuries? Q3: Does the social media strategy conduct effectively today?

After summarizing the answers of the experts, all the interviewers agree that the traditional social work actions and clinical methods aren't enough to resolve the millions of psychological injuries in China today, there must be a Marco-intervention strategy and social media is an actual choice. For example, F tells that there are certain encouraging policies to welcome social strength to play an active role in the public health program such as the online volunteer service. And there are thousands of psychologists setting up online counseling services to run businesses. This online service is a kind of social media strategy of B to C service. Experts recommend that social media as a new and open platform that can be effectively utilized to help more psychological injuries through online social media community interactive communication. ABCDEG advise that the government set up a new policy to encourage the cooperation between foundations and social organizations to establish more influential social media APP to welcome more psychologist, social worker, injuries, and other volunteers who could interact freely, safely, efficiently, and effectively.

### Relevant Policies on the Media

From the literature and expert interviews, it finds that although traditional social work strategies can play helpful roles in dealing with psychological injuries, these strategies also have many disadvantages, such as the low efficiency of case work, the slow progress of group work, and the higher cost of community social work. We are concerned about the increasing number of people who have suffered or are vulnerable to psychological harm. Social-macro strategies must be applied to develop useful and feasible approaches to address psychological injury in social work. Then, media intervention is a way to address social problems and issues. The media is undoubtedly the best platform on which to influence public opinion and transmit information. At the same time, the rapid development of new media is not only one of the reasons for the psychological problems of most groups but also a key channel of psychological relief and counseling because of its ability to rapidly transmit information and knowledge to an extensive audience (16).

At present, the scale of Chinese internet users is the largest in the world, and the internet penetration rate exceeds the global average. How policies are formed and transmitted via the internet and online psychological counseling is conducted is changing daily. Therefore, the policies described in this article promote the psychological intervention of social work via social media applications, including new media such as Weibo, WeChat, and specific apps. We know that policies controlling the media control are becoming more regulative and institutional. A series of media policies have been put in place to encourage their development and monitoring. The details of the relevant policies are shown in Table 1.

**Table 1:** Media policies issued by the central government.

**Serial number**	**Time**	**Policy name**	**Introducing department**
1	March 2002	“Opinions on Further Strengthening Internet News Propaganda and Information Content Management”	General Office of the Central Committee of the Communist Party of China, General Office of the State Council
2	February 2005	“Administrative Measures for the Recordation of Noncommercial internet Information Services”	Ministry of Information Industry
3	January 2017	“Opinions on Promoting the Healthy and Orderly Development of Mobile internet”	General Office of the Central Committee of the Communist Party of China, General Office of the State Council
4	May 2017	“Internet News Information Service Management Regulations”, “Internet News Information Service License Management Implementation Rules”, “Internet Information Content Management Administrative Law Enforcement Procedures Regulations”	National Internet Information Office
5	November 2018	“Regulations on the Security Evaluation of internet Information Services with Public Opinion Attributes or Social Mobilization Capabilities”	National Internet Information Office
6	December 2019	“Regulations on the Ecological Governance of Network Information Content”	National Internet Information Office

In 2008, the State Council issued the “Opinions of the State Council on Policies and Measures to Support the Post-Wenchuan Earthquake Recovery and Reconstruction”, which proposed the policy of “one side in trouble, eight sides supporting, self-reliance and hard work”, combining state support, social assistance, and production self-help (17). The Ministry of Civil Affairs issued the “Guidance on Supporting and Guiding Social Forces in Disaster Relief Work” on October 8, 2015, and for the first time, the participation of social forces in disaster relief work was included in the government's normative system (18). In 2014, China moved from a “big network country” to a “strong network country” in a year when cyberspace became clearer, positive network energy became more enormous, and China began to enter the “new normal” of the Internet (19). The increasing popularity of mobile Internet provides a platform and basis for preventing and solving psychological problems. In 2015, the General Office of the State Council issued, and the Health and Family Planning Commission and other departments jointly issued, the Notice of National Mental Health Work Plan (2015–2020), which included mental health work as an important initiative to protect and improve people's livelihood and strengthen and innovate social management, and was included in the overall plan of national economic and social development (20). In 2018, the General Office of the State Council issued the Opinions on Promoting the Healthy and Orderly Development of New Media for Government Affairs, pointing out that new media for government affairs is an important channel for the Party and the government to contact the masses, serve the masses and unite the masses in the mobile Internet era, an important means to accelerate the transformation of government functions and build a service-oriented government, an important position to guide online public opinion and build clear cyberspace, and an important way to explore new modes of social governance and It is an important way to explore new modes of social governance and improve social governance capacity (21). In 2020, in the face of the sudden new coronavirus pneumonia epidemic, in early March, the National Health and Health Commission and the Ministry of Civil Affairs jointly issued a notice requiring the strengthening of psychological assistance and social work services for those infected with new coronavirus pneumonia, those isolated and front-line workers in the prevention and control of the new coronavirus pneumonia epidemic; on March 18, the State Council issued the “New Coronavirus Pneumonia Epidemic Psychological Relief in the Joint Prevention and Control Mechanism Work Program”, requiring relevant departments to provide care, social support, psychological guidance, and other services according to the characteristics and psychological service needs of different groups of people (22).

Meanwhile, online ethics have made it much more convenient for professional social media experts to work with psychological injuries (23). We can take advantage of social media to support the resolution of psychological injury problems.

Because of the close relationship between social media to human social life and its immediacy, different groups will be affected at this time. To a large extent, social media can reflect social reality and people's thoughts, values, emotions, and psychological states. Whether it is a certain stage in the human life cycle or the psychological problems caused by social factors, the internet offers the opportunity for issues to be widely discussed and even resolved through social media. Therefore, social media is an effective way to carry out social work, influence public opinion, and gather support. At present, common public opinions in response to psychological problems include rumor-based posts on public sites, doubtful public sentiments, help-seeking queries, supervisory input, and complex psychological discussions (24). Social workers can use public dialogue on social media platforms to decipher rumors, spread truth, and guide the correct values and ideas about life and the world; they can resolve doubts and provide more practical access to complete social supervision and social accountability for psychological injuries.

## Results

The literature scoping shows that we can do a lot to relieve the psychological injuries in social work through traditional case work, group work, and community work, but we should also renew the strategy of employing social media as a means of healing psychological injuries in social work.

Semi-structured expert interviews show that we are facing a more regulated social media policy environment in China. Using social media to help psychological injury in social work will require more effective and practical strategies to reach the huge majority of Chinese people, including teenagers and elderly individuals, who carry smartphones. Interviews indicate a need for more public and private funds to support the social media strategy regarding social work.

## Discussion

There is a common consensus on the effective function of social media in addressing psychological injury in social work, Studies have shown that social media have a positive effect on the prevention, improvement, and treatment of psychological problems. Taking the college student group as an example, Feng Dan argues that new media has the advantages of timeliness and interactivity, which enriches the content and enhances the effectiveness of moral education in colleges and universities (25), and Song Yueqiang argues that the development of new media has, to a certain extent, promoted the development of peer psychological guidance and facilitated the psychological guidance work for some students (26). Chen Jin and Chen Wenjie's article specifically refine the advantages into three aspects: opening up the horizons of the caseworkers and helping them to improve and perfect their psychological mechanisms; expanding the activity space and coverage of mental health education work, which can also enhance the early warning function of mental health education; and facilitating the popularization of mental health education knowledge and creating a general environment for mental health education (27). These are the advantages of social media for college student groups in solving psychological problems. Facing broader social groups, such as citizens affected by epidemics, scholars such as Peng Zongchao and Huang Hao believe that media advocacy is very important for timely and effective prevention and response to the uncertain risk of unexplained infectious diseases (28). Li Jingxiu suggests that full media creates a “democratic” discourse that helps to release the negative emotions of audience opinion promptly, alleviate social conflicts, and promote harmony (29).

**Figure 1:**
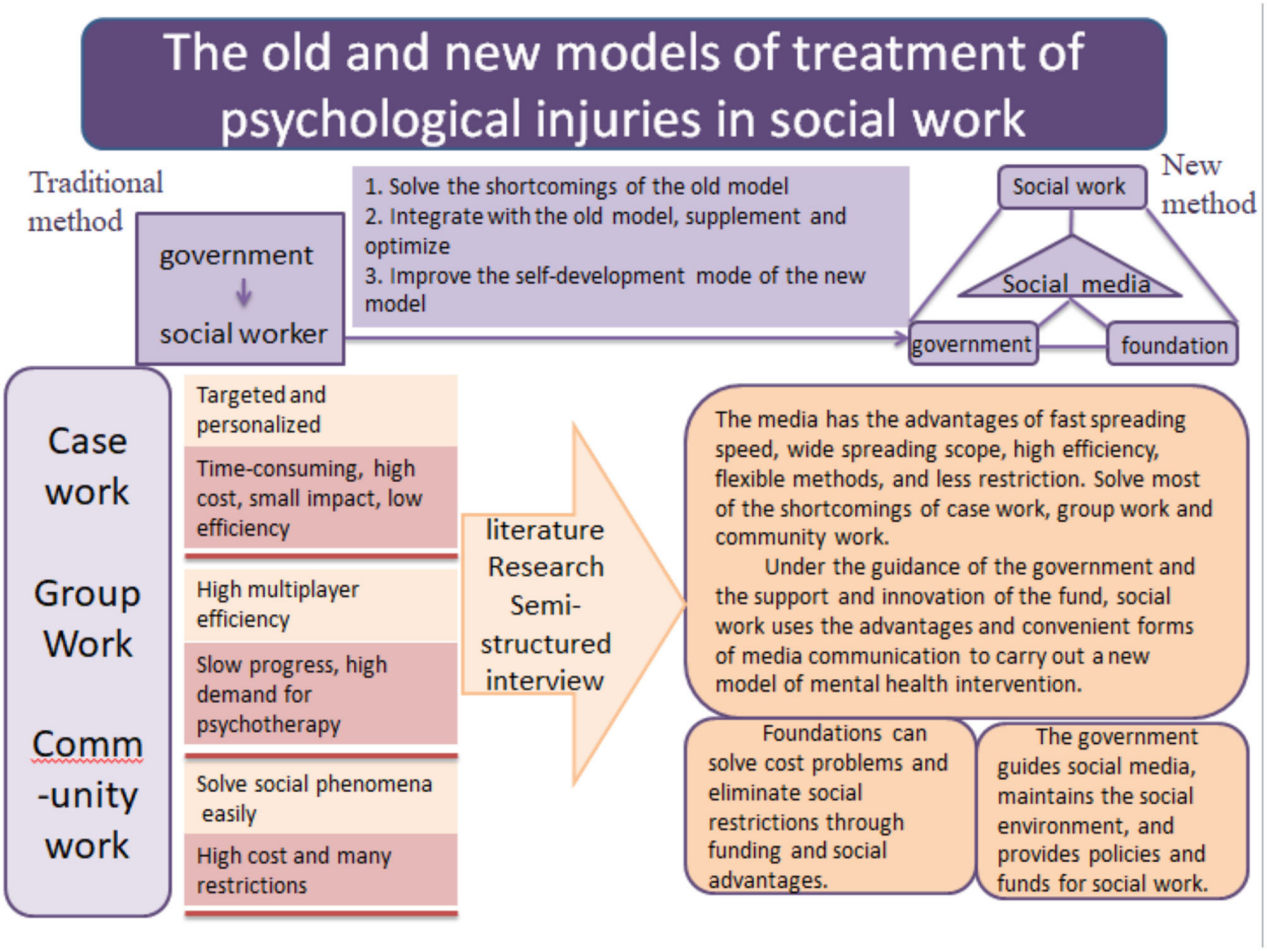
The new mechanism of social work on psychological injury.

Next, we should find better strategies to push social media into a stronger role in dealing with psychological injuries in an increasingly open society. In addition to influencing public opinion, we might look to the internet to seek funding from specific charities. Social media support is a strategy of public opinion advocation and persuasion, and it can do much in recognizing and diminishing panic about the psychological injury. Meanwhile, funding assistance is a financial tool that can cover social media strategies and traditional social work methods with a sufficient budget. Therefore, we should make full use of social media support and financial assistance to modify the traditional model into a new one to help more people with psychological injuries avoid harm and suffering. The details are shown in Figure 1.

### Creating Influencers in the Public Opinion Environment

The similarity is an important factor that enables information dissemination to resonate, stimulate discussion, and promote development, is the empathy among users of social media platforms. When a social event becomes a hot topic and attracts the attention of many people, the event must be somehow related to the interests of the majority. However, the level of understanding and discriminating ability of each group and individual is different, and most people do not have a clear direction and thorough insight. Thus, the guidance of opinion leaders or influencers is extremely important. It can be said that the ability of opinion leaders to disseminate information independently and effectively across social networks is vital. They can gather people who were originally in a state of dissociation based on connections hidden in the social network and bring them into the conversation. In the social network, the entire environment becomes tight and orderly (30). Influencers' opinions on social media platforms can exert much influence on the common mass, including psychologically vulnerable groups.

Applying opinion leaders to the support of public opinion on mental health issues can be promoted through the mainstream media. For example, *The People's Daily, Guangming Daily*, and *the Voice of CCBC* in traditional media and their new media apps and we-media are platforms that reach an extensive audience and can be used to cultivate credibility in the minds of the public. The official account of the Central Committee of the Communist Youth League on WeChat is one group through which we can voice support, and there are similar groups with great influence such as Weibo big bloggers, famous uploaders, known scholars, and star actors that could serve to spread authoritative opinions about psychological injuries when people encounter confusing information which might harm their mental health.

### Incorporating Knowledge Into the Media to Develop Communication Advantages

In addition to swaying public opinion, the use of social media also needs to satisfy the curiosity and cognition of the public. Therefore, the content, science popularization, and explanations are particularly important. For the popularization of knowledge, the more common and effective methods are books, publications, and scientific and educational radio and television programs. These forms of content dissemination are activities supported by the national government in the interest of social awareness and development. Such forms are highly controllable and professional and have made outstanding contributions to public opinion.

However, with the development of the internet, the communication effect of traditional media has been declining, and the development of new media and convergence media has become mainstream. Therefore, we need to bring public opinion support for mental health issues to new online media, expand the spread of content, and increase the sense of participation and recognition of the public under the premise of ensuring professionalism and controllability (31). Ways with a wide range of influence include public service advertisements, short videos, Moments links, and WeChat official accounts. Through the interoperability and mission of the networks, public opinion is quickly spread to achieve the strong support of the public.

### Collaborating With the Government to Accurately Monitor Public Opinion

Traditional media has good controllability and professionalism, and new media has strong dissemination in real-time. While each has its advantages and disadvantages, learning from each other's strengths is an urgent matter. When public opinion emerged in the past, it only relied on traditional media, and after discussion and exchange, a time was chosen to explain the issue to the public. In the new media age, the rapid and violent dissemination of news and opinions is more difficult to control. It is too late to take relevant measures after dissemination, and if the reaction to the post is negative, it will also have a detrimental impact on the credibility of the individuals, organizations, and the government associated with the message. Therefore, an accurate monitoring method for public opinion information should be developed. Irrelevant information should be filtered first and then screened and reported by a professional team, and the information should be communicated to the public within the “golden 4 h” (32). This path is far from enough to rely solely on social organizations and social media. Social media and government departments must be linked.

First, we must proactively build an open and transparent government system that can review and disseminate a variety of information at the same time The interactive platform would expand the channels for collecting public opinion, and grasp the source of public opinion while establishing government credibility; second, the government needs to strengthen the study of network technology, use new media to output correct public opinion, control public opinion promptly, and support the public opinion daily. Third, the government must unite with society. The media should construct a scientific and effective public opinion dissemination system and path, to achieve timely and effective public opinion support and control; Finally, While social media is developing, because of its openness and public nature, it can also be more prone to rumors that can lead to or deepen psychological problems, and “the internet is not a place outside the law”, therefore, we must strengthen the construction of the legal system, restrict the people's excessive behavior and extreme practices by law, and clarify the scope of the law.

The funding assistance strategy is the financial guarantee in social work practice. There is always a government-funded budget to solve social problems such as psychological harm. The government budget has its limitations and *blind* spots. Therefore, seeking the support of charities to assist with the development of new strategies in treating the psychological injury is an option.

### Fund Assistance to Purchase Social Services From More Social Organizations

Financial assistance from charities to purchase social services is the most common strategy in social work (33). Before implementation of the social media project, the foundation will be appraised of policies for government-supported projects and expand the scope of services with government funds. Social workers understand the status of psychological injury. They can communicate and cooperate with clients and introduce the content of the project. The foundation will also directly contact social organizations to investigate the actual conditions of specific groups with psychological injuries. After collecting data, a service plan will be developed to provide financial support to social workers and help them establish professional relationships to complete services. Throughout the process, the government will formulate policies and provide support while the foundation will improve the supply of funds and services, organize the entire project, and promote social work organizations to improve professional capabilities, thereby forming a relatively complete and efficient social service for psychological injuries.

### Funding Assistance to Supplement the Government's *Blind Spot*

The government is a management department with a relatively low tolerance for faults, and grassroots workers will not easily accept social organizations, so the cooperation between the foundation and the government will be limited. At the same time, in underdeveloped areas, the incubation and cultivation of social organizations are lacking. When the training provided by the government is insufficient, the foundation can provide more training. Fortunately, at present, foundations with public offerings have been able to provide high-quality social organization training. Training also takes advantage of social media by adopting multiple online and offline modes to provide training services for various organizations and groups of people. For the development of projects in the field of mental health, professionals are more important than materials. Therefore, the foundation actively cultivates professional personnel so that social workers trained in this process can be carried out services, thereby maximizing the efficiency of the foundation's financial assistance.

### Funding Assistance for Activating Social Media Marketing Plans

Since the traditional social work therapy method for psychological injuries is not as effective as expected, social media support must be an alternative. However, social media marketing is not easy, and it requires funding. In present social work practice, there is no specific financial arrangement on the widely organized social media program. A social media support plan is a strategy of social marketing, and it will operate like product marketing and promotion in the business (34). Therefore, there must be a specific organization to deal with the social media plan, which will involve recruiting professional staff to devise the social media strategy and cultivating a team of social media opinion leaders to deal with different issues concerning psychological injury. In other words, social media marketing plans and actions will incur considerable economic costs, and we must find more charity funds to invest in this work.

### Funding Assistance to Establish a New Mechanism of Social Work for Psychological Injury

Traditional solutions to help those with psychological injury in social work still work and also need funds from the government and private foundations. Social media support is an additional strategy that is necessary and effective in the internet age. Charitable funds can take advantage of operational efficiency to initiate the new social media support program. Once the social media support program is operational, it can attract the participation and cooperation of governments and local social organizations. Funding assistance can act as an engine to activate social organizations, government, social workers, and charitable foundations. Therefore, a new comprehensive solution can be initiated to assist the social media support program and traditional strategies in social work. In this way, we would develop social collaboration to promote psychological health in a vulnerable and anxious society (35).

## Conclusion

There are still limitations of the social media support strategy and funding assistance in actual social life and operational processes. Public opinion control has the characteristics of far-reaching spread, strong appeal, and flexible methods, but the power of public opinion control and support has certain limitations, and public opinion support also has many unavoidable defects. The first is the unpredictability of the online public opinion problem. It is difficult to filter out public opinion keywords from the huge amount of information; the second is that the number of people participating in media rumors is too large, and it is difficult to balance the correct public opinion direction and the best time in the process to dispel rumors; Third, netizens tend to be younger and less disciplined, creating a large number of participants in cyberspace, information explosions, and management difficulties. Especially for children and young netizens with immature cognitive skills, there may be great harm caused by the internet when information cannot be relied upon. Education about the online environment is improving. However, the conflict of public opinion will increase the conflict of values. Finally, internet populism will weaken the authority of the government and the elite. Young and less educated people rely on intuition and habits to judge things and often have extreme concepts. In addition, they usually have a skeptical and negative attitude toward the government (36).

Foundations provide strong financial support for social projects, which is one of the powerful driving forces behind social progress. However, there are still many problems in the development of foundation projects. The first is that the current situation of social mentality is not optimistic. There are a small number of professional teams that can directly and efficiently carry out social services and operate well in public welfare programs. Therefore, charitable funds face hard choices and have less interest in assisting social work programs. Second, the budget from government funds is always enough to cover more social work programs, especially social media strategies. Therefore, there is a large funding gap for conducting large-scale social media marketing strategies in the internet society. Finally, charitable fund management has challenges. There are still many doubts about the credibility of foundations because of low transparency and negative publicity.

In conclusion, it is urgent to help individuals with psychological injury in social work since we are constructing a “people-centered society” in a new period of history. Although there are problems, we should deal with this issue through innovation both in policy and practice. On the whole, we should continuously encourage traditional social work strategies, but we can take measures from a macro perspective of social media support programs to help those with psychological injuries in the internet age. Furthermore, we should create new opportunities for the growth of charitable foundations and their funds to assist more traditional social work programs as well as the new social media support plan. We believe that social media support and funding assistance for treating psychological injuries in social work can move forward in modern China.

## Important Terms Explained

In the Chinese context, people often negatively define rumors, emphasizing that rumors are intentionally created information with a specific purpose and direction, while the meaning of “rumor” in the English context is not all those information that is created out of nothing or fabricated out of thin air (37). The French scholar Cap Ferré has given a neutral definition of rumor: “A rumor is a piece of information that appears and circulates in society without official public confirmation or has been officially disproved” (38).

Based on the need for research, the authors of this paper, drawing on the value-neutral position of Capulet on rumors and taking into account the actual diversity of information on social media platforms, expand the scope of rumors and define them as unofficially confirmed information that is widely spread on social media platforms in the form of modern online media and has a psychological and life impact on viewers. In this paper, we define “rumor” as an unconfirmed narrative about certain people, groups, events, and institutions that are widely spread on social media platforms in a modern online media format and have a psychological and life impact on viewers (39).

A foundation, also known as a charitable foundation, is a non-profit legal entity established following the regulations to engage in public welfare through the use of property donated by natural persons, legal entities, or other organizations (40).

## Data Availability Statement

The original contributions presented in the study are included in the article, further inquiries can be directed to the corresponding author.

## Ethics Statement

The studies involving human participants were reviewed and approved by IRB of NJU (No: NJUPSY202105003). The Ethics Committee waived the requirement of written informed consent for participation.

## Author Contributions

XH has designed the logic of the paper and put forward the new mechanism of the social media support strategy. CT has finished the literature review job. DW has made the proof writing job and given much suggestive advice to the paper. All authors contributed to the article and approved the submitted version.

## Funding

This study was supported by the Youth Project of Social Science Fund of Jiangsu Province (Grant 20SHC001).

## Conflict of Interest

The authors declare that the research was conducted in the absence of any commercial or financial relationships that could be construed as a potential conflict of interest.

## Publisher's Note

All claims expressed in this article are solely those of the authors and do not necessarily represent those of their affiliated organizations, or those of the publisher, the editors and the reviewers. Any product that may be evaluated in this article, or claim that may be made by its manufacturer, is not guaranteed or endorsed by the publisher.
